# Acute Maternal Infection and Risk of Pre-Eclampsia: A Population-Based Case-Control Study

**DOI:** 10.1371/journal.pone.0073047

**Published:** 2013-09-03

**Authors:** Caroline Minassian, Sara L. Thomas, David J. Williams, Oona Campbell, Liam Smeeth

**Affiliations:** 1 Faculty of Epidemiology and Population Health, London School of Hygiene and Tropical Medicine, London, United Kingdom; 2 Institute for Women’s Health, University College London Hospital, London, United Kingdom; Yale School of Public Health, United States of America

## Abstract

**Background:**

Infection in pregnancy may be involved in the aetiology of pre-eclampsia. However, a clear association between acute maternal infection and pre-eclampsia has not been established. We assessed whether acute urinary tract infection, respiratory tract infection, and antibiotic drug prescriptions in pregnancy (a likely proxy for maternal infection) are associated with an increased risk of pre-eclampsia.

**Methods and Findings:**

We used a matched nested case-control design and data from the UK General Practice Research Database to examine the association between maternal infection and pre-eclampsia. Primiparous women aged at least 13 years and registered with a participating practice between January 1987 and October 2007 were eligible for inclusion. We selected all cases of pre-eclampsia and a random sample of primiparous women without pre-eclampsia (controls). Cases (n = 1533) were individually matched with up to ten controls (n = 14236) on practice and year of delivery. We calculated odds ratios and 95% confidence intervals for pre-eclampsia comparing women exposed and unexposed to infection using multivariable conditional logistic regression. After adjusting for maternal age, pre-gestational hypertension, diabetes, renal disease and multifetal gestation, the odds of pre-eclampsia were increased in women prescribed antibiotic drugs (adjusted odds ratio 1.28;1.14–1.44) and in women with urinary tract infection (adjusted odds ratio 1.22;1.03–1.45). We found no association with maternal respiratory tract infection (adjusted odds ratio 0.91;0.72–1.16). Further adjustment for maternal smoking and pre-pregnancy body mass index made no difference to our findings.

**Conclusions:**

Women who acquire a urinary infection during pregnancy, but not those who have a respiratory infection, are at an increased risk of pre-eclampsia. Maternal antibiotic prescriptions are also associated with an increased risk. Further research is required to elucidate the underlying mechanism of this association and to determine whether, among women who acquire infections in pregnancy, prompt treatment or prophylaxis against infection might reduce the risk of pre-eclampsia.

## Introduction

Pre-eclampsia is a multi-system vascular syndrome of pregnancy defined by the gestational onset of hypertension and proteinuria, typically occurring after 20 weeks’ gestation. It is a major cause of maternal and perinatal morbidity and mortality worldwide, its incidence ranging between 2% and 8% in nulliparous women. [1] Despite advances in knowledge, we still have a limited ability to predict or prevent pre-eclampsia. While its aetiology is generally considered to be multifactorial, involving both maternal and placental contributions, [2] there is increasing evidence that inflammation plays a central pathogenic role. [3] Impaired vascular endothelial function, which can derive from inflammation, is evident among women prior to developing pre-eclampsia. [4] Poor placental perfusion as a result of inadequate placentation is a key inflammatory stimulus for many women with pre-eclampsia. However, any factor that provokes the maternal systemic inflammatory response, such as infection, may contribute to the overall inflammatory burden and the development of pre-eclampsia.

A growing body of evidence suggests that infection, a common cause of inflammation and of endothelial dysfunction, may be involved in the aetiology of pre-eclampsia. [5] An increased risk of pre-eclampsia associated with maternal periodontal disease has been well-documented.[6]–[8] Studies based on serological markers of chronic infections have also yielded positive findings,[9]–[14] although temporal associations in these studies are uncertain.

Acute maternal infections such as urinary tract infection (UTI) may also play a role in pre-eclampsia, possibly by amplifying the maternal systemic inflammatory response. A meta-analysis of observational studies examining the relationship between maternal infections and pre-eclampsia [5] reported a summary odds ratio for pre-eclampsia of 1.57 (95% CI 1.45–1.70) in women with UTI in pregnancy. However, there was marked heterogeneity between studies and results were inconsistent. Findings from two more recent studies are conflicting: one, a large population-based cohort study [15] reported an increased risk of pre-eclampsia among women with maternal UTI, while a case-control study found no association. [16] Factors such as the timing of infection in relation to pre-eclampsia were not investigated in these studies, and the findings may have been confounded by renal disease, or biased by increased ascertainment of UTI in pregnancy, particularly among women at risk of pre-eclampsia. Data on the effects of other acute maternal infections are lacking. Thus a clear role for acute infection in the aetiology of pre-eclampsia has not been established.

The large sample size afforded by the UK General Practice Research Database (GPRD) provided a unique opportunity to address these issues. We assessed the gestational onset of UTI, as well as respiratory tract infection (RTI), and maternal antibiotic prescriptions (a likely proxy for infection). Examining the role of antibiotic prescriptions and infections in different organ systems would, if positive, suggest that the effect of acute infection on the risk of pre-eclampsia is generic and not specific to one type of infection.

## Methods

### The General Practice Research Database

The GPRD is an electronic UK population-based primary care database. Established in January 1987, it holds anonymised longitudinal patient records, routinely recorded as part of patients’ normal care, for over 10 million patients registered to over 600 general practices. More than 98% of the UK population are registered with a general practitioner (GP) and practices contributing to the database are representative of practices throughout the UK. [17] In addition to being a rich data source, the GPRD has high data validity. [18] Each participating practice is assigned an “up-to-standard” date indicating when data recording complied with specific quality measures (based on an assessment of the completeness, continuity and plausibility of data). Data are subject to ongoing evaluation, verification and validation procedures to ensure they are research-quality [19].

### Ethics Statement

The electronic health records used for this study comprised data from the Full Feature GPRD obtained under licence from the UK Medicines and Healthcare Products Regulatory Agency. GPRD data are used extensively for public health research, and the GPRD has stringent procedures for maintaining confidentiality of personal data. All data provided to researchers are anonymised to ensure that individual patients cannot be identified. In addition, patients have the right to opt out from the use of their anonymised data. The use of these data for this study was approved by the Independent Scientific Advisory Committee of the GPRD (protocol 07_094) and by the London School of Hygiene and Tropical Medicine Ethics Committee (application number 5283).

### Study Design and Participants

We used a matched nested case-control study design to examine the association between acute maternal infections and pre-eclampsia. Participants were derived from a source population of all female patients registered with a practice contributing to the GPRD between 1^st^ January 1987 and 31^st^ October 2007 inclusive and who had a pregnancy during this period.

#### Eligibility criteria

Because pre-eclampsia usually develops in the later stages of pregnancy, a woman must have completed her pregnancy to have the opportunity to become a case. Therefore, only women with a documented completed pregnancy, defined as an end-of-pregnancy record indicating the woman had delivered a live birth or stillbirth (e.g. “birth details”), or was soon to deliver (e.g. “antenatal 37 week examination”), were potential candidates. Pregnancies resulting in miscarriage or termination were not included since they were likely to end before a woman had reached the required gestational age to be at risk of being diagnosed with pre-eclampsia. As pre-eclampsia is much more common in nulliparous women, [20] we restricted the study population to women with a first documented completed pregnancy during the study period.

Women aged at least 13 years at delivery and whose data throughout the gestational period (from conception to delivery) were within up-to-standard follow-up were eligible for inclusion. The follow-up criterion helped ensure that diagnoses and events pertaining to the pregnancy were captured. Clinical entries in the data were coded using the Oxford Medical Information System (OXMIS) and Read coding system. We selected as potential cases all those with a clinical diagnosis of pre-eclampsia, defined as a Read/OXMIS code for pre-eclampsia, eclampsia or the severe pre-eclampsia variant HELLP syndrome (hemolysis, elevated liver enzymes, low platelets), in their first documented completed pregnancy. We selected as potential controls a random sample of eligible women with no diagnosis of pre-eclampsia anywhere in their medical data. This ensured controls had no pre-eclampsia in their first documented completed pregnancy, and no history of pre-eclampsia possibly relating to an earlier (unrecorded) pregnancy.

#### Exclusions

**Figure 1:**
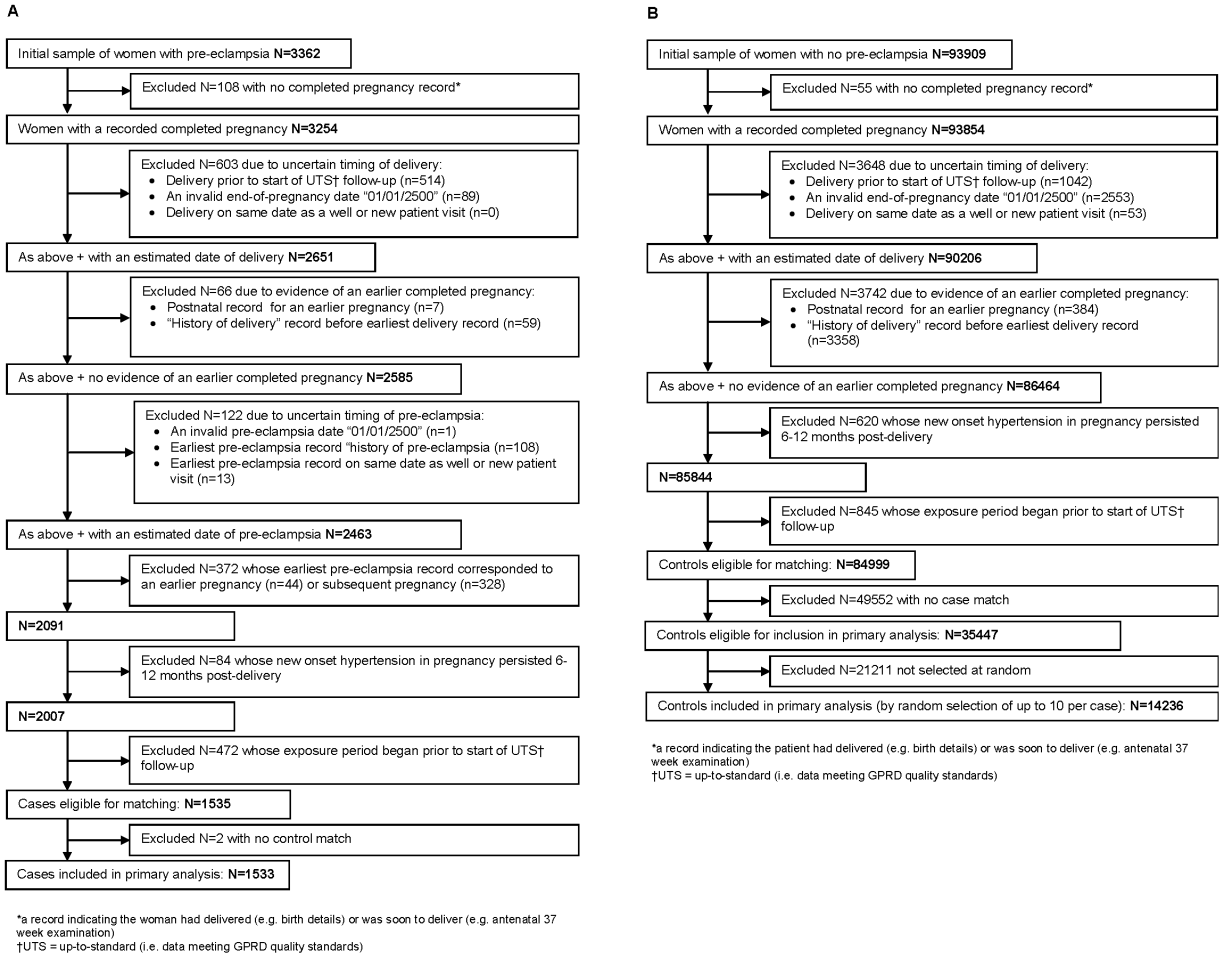
Identification of study participants included in the primary analysis: A) cases (n = 1533); B) controls (n = 14236).

To help ensure cases and controls were primiparous, we excluded women with evidence for an earlier completed pregnancy (e.g. a record of “previous caesarean section” before their earliest delivery record). To distinguish cases of pre-eclampsia from women with essential or secondary hypertension which became clinically apparent during pregnancy, we excluded women with no evidence of high blood pressure until pregnancy but whose hypertension did not resolve six to 12 months post-delivery. The identification of cases and controls for inclusion in the study is illustrated in **Figure 1**.

#### Matching

Cases were matched with controls on GP practice to allow for variability in recording and prescribing habits between practices, and on year of delivery (an absolute difference of up to 12 months between cases’ and controls’ estimated delivery dates) to ensure they were contemporaneous. All possible case-control matches were identified and up to ten controls per case were selected at random without replacement. Although any additional gain in power is minimal if the case-control ratio exceeds 1∶4, the number of eligible controls exceeded the number of cases by more than twenty-fold: thus, increasing the control-per-case ratio beyond four posed no additional cost or effort in data collection.

### Dating Pregnancies

In the absence of systematically recorded information on the exact timing of pregnancy, we used information from antenatal records indicating gestational age, delivery records indicating the number of days or weeks postnatal, and recorded estimates of the expected date of delivery and first day of a woman’s last menstrual period (LMP) to obtain our best estimates of the start and end of each woman’s completed pregnancy. We estimated the timing of trimesters adopting a common convention: first trimester (first day of LMP to 13 weeks), second (weeks 14 to 26), and third (week 27 to delivery).

### Exposures

The exposure period for each participant began on the first day of LMP and ended at the index date, defined as the date of pre-eclampsia diagnosis (for cases). For controls, the index date was the date they reached the same gestational age as their matched case at pre-eclampsia diagnosis. This was to ensure the duration of the exposure period for cases and their matched controls was comparable.

We extracted data on Read/OXMIS codes for acute UTIs (manifest as asymptomatic bacteriuria, cystitis, or pyelenephritis) and RTIs (excluding non-specific or minor upper RTIs and symptoms such as sore throat), and on antibiotic drug prescriptions over the exposure period. When a woman had more than one record of infection or antibiotic prescription, a minimum of 29 days between records of the same type was required for these to be considered distinct episodes of infection (rather than repeat records for the same infection).

#### Potential confounders

Data on the following potential risk factors were extracted: maternal age; pre-gestational renal disease, diabetes, hypertension and asthma; multifetal gestation; pre-pregnancy body mass index (BMI); maternal smoking (a known protective factor); previous early pregnancy loss; and assisted reproductive technology (ART), defined as in vitro fertilization and related techniques (including gamete intrafallopian transfer and embryo transfer). To address the possibility that some infections may be more likely to be recorded among women who consult with their GP more frequently, we measured the number of consultations and duration of follow-up each woman had prior to pregnancy. This allowed comparison of cases’ and controls’ pre-pregnancy consultation behaviour.

### Statistical Analysis

We used multivariable conditional logistic regression to estimate odds ratios (ORs) and 95% confidence intervals for pre-eclampsia comparing pregnant women exposed and not exposed to each type of infection or to antibiotic prescriptions. The primary analysis assessed the effect of each exposure at any time during the exposure period. Subsequent analyses explored the effects of increasing episodes of infection over the exposure period. Potential confounding factors associated with pre-eclampsia in crude analyses were assessed in more complex models and retained if they made an appreciable difference to the infection (or antibiotics) OR. Maternal age (controlled for in five-year age groups) and pre-existing renal disease were included in all models a priori. Likelihood ratio tests were used to assess statistical significance.

To reduce the possibility of misdiagnosis of UTI among women with pre-eclampsia due to identification of proteinuria, we repeated the analyses for UTI and antibiotics among cases who developed pre-eclampsia in the third trimester and their matched controls, and assessed the effect of exposure to UTI or antibiotics in the first and second trimesters only versus no exposure at any time in pregnancy. These third trimester pre-eclampsia cases were unlikely to have proteinuria detected during the first two trimesters, thus minimising the potential for such misclassification.

In sensitivity analyses, we excluded the following: women aged less than 18 years as their pregnancy outcomes and underlying risk profile may differ from older women; women with pre-existing hypertension, to rule out the possibility of misdiagnosis of pre-eclampsia; controls with new onset hypertension during pregnancy which resolved shortly after delivery, as they may have had pre-eclampsia even in the absence of a clinical diagnosis; and ART pregnancies which may have a higher risk of developing pre-eclampsia. To reduce the possibility that events we identified throughout the exposure period (e.g. infections) may have referred to past diagnoses that were recorded retrospectively within the first few months after a patient joined a practice, we extended the up-to-standard follow-up criterion to include only women with at least six months up-to-standard follow-up prior to conception. To address the possibility that pre-eclampsia diagnoses made in earlier years were less exact, we restricted our analyses to pregnancies in year 2000 onwards following publication of the first recommended consensus definition of pre-eclampsia. [21] Finally, to assess whether the effect of infection differed according to the timing of onset of pre-eclampsia, or the severity, we conducted separate analyses for cases with early-onset (<34 weeks’ gestation) versus late-onset (≥34 weeks’ gestation) pre-eclampsia, and for cases with documented severe pre-eclampsia, eclampsia or HELLP syndrome.

Data were analysed using Stata, release 12 (StataCorp., College Station, Texas).

## Results

Data were obtained on all women with a clinical diagnosis of pre-eclampsia during the study period (3362 potential cases) and a large random sample of women who had a pregnancy during this period and no recorded diagnosis of pre-eclampsia (93909 potential controls). After applying the eligibility, exclusion and matching criteria, 1533 pre-eclampsia cases and 14236 controls were included in the primary analysis (see **Figure 1**). The commonest reason for not being eligible was uncertainty about the timing of pregnancy. **Table 1** summarizes the demographic and risk profile of study participants. The median gestational age of cases at pre-eclampsia diagnosis was 38.1 weeks (interquartile range (IQR) 34.9 to 39.9 weeks), and most cases (79.5%) were late-onset (≥34 weeks’ gestation). The majority of pre-eclampsia diagnoses were non-specific regarding severity (47.3%) or mild (32.0%); the remaining 20.7% were severe pre-eclampsia, eclampsia or HELLP syndrome. Cases and controls were of similar age at delivery (median 28.3 years for cases; 28.2 years for controls) and shared similar pre-pregnancy consultation behaviour (median 11 consultations over 2.5 years). A higher proportion of cases were overweight or obese (30.3%) compared to controls (20.4%), and cases were less likely to smoke (18.5%) than controls (23.3%).

**Table 1:** Characteristics of study participants.

Characteristic n (%)	Cases (N = 1533)	Controls (N = 14236)
**Maternal age at delivery (yrs)**		
<20	132 (8.6)	1470 (10.3)
20–24	340 (22.2)	2846 (20.0)
25–29	478 (31.2)	4492 (31.6)
30–34	406 (26.5)	3803 (26.7)
35–39	146 (9.5)	1348 (9.5)
40–44	29 (1.9)	239 (1.7)
≥45	2 (0.1)	38 (0.3)
*median, IQR*	*28.3, 23.9–32.3*	*28.2, 23.9–32.1*
**Pre-pregnancy BMI (kg/m^2^)**		
<18.5 (underweight)	26 (1.7)	526 (3.7)
18.5–25 (normal)	620 (40.4)	6618 (46.5)
25–30 (overweight)	272 (17.7)	1959 (13.8)
30+ (obese)	192 (12.5)	946 (6.7)
unknown	423 (27.6)	4187 (29.4)
*median, IQR*	*24.1, 21.6–27.9*	*22.7, 20.7–25.6*
**Smoking status in pregnancy**		
non-smoker	834 (54.4)	6680 (46.9)
ex-smoker	166 (10.8)	1476 (10.4)
current smoker	283 (18.5)	3311 (23.3)
unknown	250 (16.3)	2769 (19.5)
**Practice level socioeconomic status** a		
IMD score *[median, IQR]*	*16.2, 8.7–30.1*	*16.3, 8.4–30.2*
**Patient level socioeconomic status**		
IMD score *[median, IQR]*	*14.3, 8.4–25.7*	*14.8, 8.3–26.4*
unknown	744 (48.5)	6734 (47.3)
**Pre-existing hypertension**	161 (10.5)	875 (6.2)
**Pre-existing renal disease**	4 (0.3)	25 (0.2)
**Pre-existing diabetes**	28 (1.8)	166 (1.2)
**Pre-existing asthma**	291 (19.0)	2509 (17.6)
**Previous miscarriage or termination**	298 (19.4)	2869 (20.2)
**Multiple pregnancy**	25 (1.6)	121 (0.9)
**ART pregnancy**	11 (0.7)	84 (0.6)
**Consultations with GP pre-pregnancy** *[median, IQR]*	*11, 4–27*	*11, 4–24*
**UTS follow-up pre-pregnancy (yrs)** *[median, IQR]*	*2.4, 0.9–5.1*	*2.5, 1.1–5.3*

Abbreviations: IMD = Index of Multiple Deprivation score based on practice post-code (practice level socioeconomic status) or patient post-code (patient level socioeconomic status). The higher the score the greater the deprivation. UTS = up-to-standard (i.e. data meeting GPRD quality standards). ART = assisted reproductive technology.

a matching variable.

During their first completed pregnancy, 528 (34.4%) cases and 4110 (28.9%) controls were prescribed an antibiotic drug, 182 (11.9%) cases and 1376 (9.7%) controls had one or more UTI, and 77 (5.0%) cases and 781 (5.5%) controls had one or more RTI. The timing of each exposure by pregnancy trimester is shown in **Table 2**. Less than half of women with an antibiotic prescription in pregnancy had a record of UTI or RTI (42.8% of cases; 44.3% of controls).

**Table 2:** Frequency of maternal infection or antibiotic treatment in pregnancy and by pregnancy trimester.

Exposure in pregnancy^a^ n (%)	Cases (N = 1533)	Controls (N = 14236)
**Antibiotic treatment**		
Any time in pregnancy	528 (34.4)	4110 (28.9)
First trimester	221 (14.4)	1684 (11.8)
Second trimester	238 (15.5)	1952 (13.7)
Third trimester	203 (13.2)	1520 (10.7)
**Urinary tract infection**		
Any time in pregnancy	182 (11.9)	1376 (9.7)
First trimester	64 (4.2)	463 (3.3)
Second trimester	81 (5.3)	606 (4.3)
Third trimester	57 (3.7)	487 (3.4)
**Respiratory tract infection**		
Any time in pregnancy	77 (5.0)	781 (5.5)
First trimester	31 (2.0)	293 (2.1)
Second trimester	29 (1.9)	307 (2.2)
Third trimester	24 (1.6)	218 (1.5)

Note some women had more than one exposure in the same (or in another) trimester.

a any time from 1^st^ day of last menstrual period (LMP) to index date (for cases this is the date of pre-eclampsia, for controls this is the date they reached the same gestational age as their matched case at the case’s index date).

Crude and adjusted ORs for the association between maternal infection and pre-eclampsia are summarized in **Table 3**. Antibiotic prescriptions (adjusted OR 1.28; 1.14–1.44) and UTI (adjusted OR 1.22; 1.03–1.45) in pregnancy were associated with an increased risk of pre-eclampsia after controlling for maternal age; pre-gestational renal disease, diabetes and hypertension; and multifetal gestation. We found no evidence for confounding by prior early pregnancy loss. Findings were virtually identical when we addressed the potential for differential misclassification of UTI due to detection of proteinuria in cases by repeating analyses after confining the exposure window for UTI and antibiotics to the first two trimesters and excluding cases (n = 41) with very early onset pre-eclampsia prior to the third trimester: adjusted OR for antibiotics 1.26 (1.11–1.43) and UTI 1.22 (1.01–1.49). Further adjustment for pre-pregnancy BMI and maternal smoking among individuals with BMI and smoking data (1048 cases and 7216 controls) made no material difference to our findings (**Table S1**).

**Table 3:** The association between maternal infection and pre-eclampsia: crude and adjusted odds ratios for matched cases (n = 1533) and controls (n = 14236).

Exposure in pregnancy^a^	Matched crude OR (95% CI)	Matched adjusted^b^ OR (95% CI)
Antibiotic treatment	1.29 (1.15–1.44)	1.28 (1.14–1.44)
Urinary tract infection	1.23 (1.04–1.46)	1.22 (1.03–1.45)
Respiratory tract infection	0.91 (0.71–1.15)	0.91 (0.72–1.16)

a any time from 1^st^ day of last menstrual period (LMP) to index date (for cases this is the date of pre-eclampsia, for controls this is the date they reached the same gestational age as their matched case at the case’s index date).

b ORs adjusted for maternal age; pre-gestational hypertension, diabetes and renal disease; and multifetal gestation. In addition, ORs for UTI and RTI are mutually adjusted for.

Women exposed to RTI in pregnancy were more likely to have pre-existing asthma (29.5%) than women not exposed to RTI (17.1%), and were more likely to smoke (28.6%) than those not exposed (22.5%). We found no association between RTI and pre-eclampsia in either the crude or adjusted analyses. The inclusion of pre-existing asthma to our model did not alter the RTI OR.

The frequency distribution of the number of episodes (none, one, more than one) of infection or antibiotic treatment is shown in **Table S2**. No evidence of a dose-response association was demonstrated (data not shown). Consistent with the primary analysis, we observed an increased risk of both early- and late-onset pre-eclampsia associated with maternal antibiotics prescriptions and UTI, with no evidence for a clear difference between these two sub-groups (**Table S3**). We conducted additional sensitivity analyses as outlined in the methods. Each of these analyses yielded estimates similar to those obtained in our primary analysis (**Table S4**).

## Discussion

Our study has shown that women who acquire UTI during pregnancy, and women prescribed antibiotics during pregnancy (a likely proxy for acute infection) are at an increased risk of pre-eclampsia. The increased risk of pre-eclampsia developing in the third trimester following UTI or antibiotic prescriptions in the first two trimesters suggests that acute maternal infection may play a role in the pathogenesis of pre-eclampsia. However, we found no evidence for an increased risk of pre-eclampsia among women who acquire RTI during pregnancy.

A major strength of our study is the use of a population-based cohort of women from which we selected all cases of pre-eclampsia in a first completed pregnancy and a random sample of primiparous controls without pre-eclampsia. Our nested case-control design avoids the common problem of selection bias inherent in many case-control studies, particularly those in which the base population giving rise to the cases is less well-defined. Matching on GP practice allowed for variability in recording and prescribing habits between practices, and helped ensure that cases and controls were comparable on a range of socio-economic and environmental indicators. The additional criterion of allowing no more than 12 months between case and control delivery dates ensured pregnancies within matched sets were contemporaneous.

Another strength of this study is that we were able to include data on a substantial number of well-known risk factors for pre-eclampsia, some of which, most notably renal disease and diabetes, were not accounted for in previous studies of UTI and pre-eclampsia. [5], [15], [16] The associations with UTI and antibiotics persisted even after adjustment for maternal age; pre-existing renal disease, diabetes and hypertension; and multifetal gestation. We cannot exclude the possibility of residual confounding if disease risk factors were not recorded for some women; for example, the low prevalence of pre-existing renal disease among cases (0.3%) and controls (0.2%) suggests ascertainment of renal disease may be limited to the more severe end of the disease spectrum. However, this is unlikely to be a major concern since it is the more severe disease (stages 3–5) which predisposes to pre-eclampsia, rather than mild renal disease. [22] Missing information on maternal smoking and pre-pregnancy BMI limited our ability to assess their effects in the entire study population. Nevertheless, additional adjustment for BMI and smoking made no material difference to our findings. The similar pre-pregnancy consultation behaviour of cases and controls suggests our findings are unlikely to be explained by possible increased ascertainment of infection among cases due to differential health-seeking behaviour.

We were able to restrict the study population to women in their first documented completed pregnancy in their primary care record, and excluded women with evidence of an earlier (unrecorded) completed pregnancy, for example, a record indicating parity>0 prior to the earliest delivery record. While not guaranteeing that this was their first ever completed pregnancy, it was likely to be the first for a large majority. In addition, because the risk of infections is unlikely to have a strong relationship with parity, the scope for confounding by parity is limited. A further advantage of our approach is that it reduced the potential for confounding by change in paternity or by inter-pregnancy interval among multiparas [23].

While there is no universal agreement on the definition of pre-eclampsia, [2] a diagnosis has major consequences for a pregnant woman and is unlikely to be recorded speculatively. In 2000, the National High Blood Pressure Education Program Working Group developed diagnostic criteria for pre-eclampsia, [21] recommended in the American College of Obstetricians and Gynecologists practice guidelines for diagnosing pre-eclampsia. [24] To improve the validity of our case definition we excluded from the primary analysis women whose new onset hypertension in pregnancy did not resolve following delivery, in line with this consensus definition. While we cannot rule out the possibility of misclassification of pre-eclampsia, this criterion helped distinguish cases of pre-eclampsia from women with essential or secondary hypertension that became clinically apparent during pregnancy. Furthermore, restricting our analyses to pregnancies (and hence pre-eclampsia diagnoses) in year 2000 onwards made no material difference to our findings.

We used information from antenatal, perinatal and postnatal records to estimate the date of conception, delivery, and trimesters for all primiparous pregnancies. Although the timing may be inexact, the same method was used for dating case and control pregnancies, so any imprecision is likely to be non-differential. The main consequence for this study is that some infections early in pregnancy may have been missed if they were misclassified as occurring prior to conception. We used the date of diagnosis (or antibiotic prescription) rather than the date of onset of infection. However, the majority of patients, even with upper RTIs, attend their general practitioner within three days of onset. [25] This small degree of imprecision is unlikely to materially affect our results.

We recognize that not all infections lead to a GP consultation, so some may not have been recorded. However, such infections are more likely to be minor or asymptomatic; those severe enough to cause systemic inflammation are more likely to result in a consultation and be detected. We cannot rule out the possibility that the observed associations with maternal UTI and antibiotic prescriptions may in part be attributed to increased ascertainment of infections among women with problematic pregnancies. However, unlike previous studies we also investigated the effect of acute RTI; the null effect we observed for RTI suggests that ascertainment bias is unlikely.

Possible misclassification of UTI among women with pre-eclampsia due to detection of proteinuria was addressed by restricting the exposure period for infection to the first two trimesters, prior to the onset of pre-eclampsia in the third trimester. The resulting effect estimates for both UTI and antibiotics were virtually identical to those obtained in the primary analysis.

In our study, more than half of women with an antibiotic prescription in pregnancy had no urinary or respiratory indication, a finding which has previously been noted in primary care data. [26] While some antibiotics might have been prescribed prophylactically against recurrent infections, this is likely to be a small minority: the majority will be given for acute infections such as UTIs which are particularly common in pregnancy. [27] Nevertheless, we cannot exclude the possibility that our finding of an antibiotic effect may reflect an association with the drugs themselves rather than an association with acute infection (the main indication for their use).

Various hypotheses have been proposed to explain the mechanism by which maternal infection may be associated with pre-eclampsia. A key feature of pre-eclampsia is the greater systemic inflammatory response of women who develop the syndrome compared to women who have normal pregnancies, [28] which suggests that inflammation may play an important role in the pathogenesis. Acute infections such as UTI are an important source of inflammation. Thus, the underlying mechanism of infection may be indirect, by enhancing the maternal systemic inflammatory response. It may also include direct effects of infectious agents increasing the risk of acute uteroplacental atherosis, [29] resulting in increased systemic inflammation and vascular endothelial dysfunction preceding the clinical onset of pre-eclampsia. Although the exact mechanism of the association is uncertain, our finding of an increased risk of pre-eclampsia associated with both acute UTI and maternal antibiotic prescriptions lends support to the hypothesis that maternal infection may play a pathogenic role. The relatively few individuals with more than one episode of infection limited our ability to reliably examine a dose-effect.

The absence of an association with RTI in our study is intriguing and warrants further investigation, although it does not preclude the possibility of a generic effect of infection on pre-eclampsia risk. Our adjusted analyses suggest this finding is unlikely to be explained by the higher prevalence of maternal smoking (known to protect against pre-eclampsia) among women with RTI. However, it may in part be due to incomplete ascertainment of RTI consultations. We excluded from our definition of RTI any non-specific RTI diagnoses (e.g. a record of “Acute respiratory infection” or “Respiratory tract infection”) as it was unclear whether these were minor upper RTIs or more severe lower RTIs. We expect any such non-differential misclassification would lead to an underestimate of effect. The increased risk of pre-eclampsia we observed among pregnant women with UTI and with antibiotic prescriptions (a proxy for any acute maternal infection, including but not restricted to UTI or RTI) is consistent with a generic effect, suggesting as it does that the effect may not be specific to one type of infection.

We conclude that acute maternal UTI and antibiotic drug prescriptions in pregnancy (a likely proxy for infection) are associated with an increased risk of pre-eclampsia. Further research is required to elucidate the underlying mechanism of this association and to determine whether, among women who acquire infections in pregnancy, prompt treatment or prophylaxis against infection might reduce the risk of pre-eclampsia.

## Supporting Information

Table S1 **The association between maternal infection and pre-eclampsia: crude and adjusted odds ratios for matched cases and controls with data on pre-pregnancy BMI and smoking status in pregnancy (n = 1048 cases; n = 7216 controls).**
(DOCX)

Table S2 **Episodes of exposure to maternal infection or antibiotic treatment in pregnancy.**
(DOCX)

Table S3 **Adjusted odds ratios for early-onset (<34 weeks’ gestation) and late-onset (≥34 weeks’ gestation) pre-eclampsia.**
(DOCX)

Table S4 **Results of sensitivity analyses.**
(DOCX)
