# Supplementary material for: Acute Maternal Infection and Risk of Pre-Eclampsia: A Population-Based Case-Control Study
Source: PLoS One. 2013 Sep 3;8(9):e73047. doi: 10.1371/journal.pone.0073047 (PMC3760871; doi:10.1371/journal.pone.0073047)
Supplement: Table S1 — The association between maternal infection and pre-eclampsia: crude and adjusted odds ratios for matched cases and controls with data on pre-pregnancy BMI and smoking status in pregnancy (n = 1048 cases; n = 7216 controls). (DOCX) [file pone.0073047.s001.docx]

| **Exposure in pregnancy^a^** | **Matched crude OR (95% CI)** | **Matched adjusted^b^ OR (95% CI)** |
| --- | --- | --- |
| Antibiotic treatment | 1.23 (1.06-1.42) | 1.21 (1.05-1.40) |
| Urinary tract infection | 1.24 (1.01-1.52) | 1.24 (1.01-1.53) |
| Respiratory tract infection | 0.92 (0.68-1.25) | 0.90 (0.66-1.23) |

^a^any time from 1st day of last menstrual period (LMP) to index date (for cases this is the date of pre-eclampsia, for controls this is the date they reached the same gestational age as their matched case at the case’s index date).

^b^ORs adjusted for maternal age; pre-gestational hypertension, diabetes and renal disease; multifetal gestation; BMI and smoking. In addition, ORs for UTI and RTI are mutually adjusted for.
