# Supplementary material for: Acute Maternal Infection and Risk of Pre-Eclampsia: A Population-Based Case-Control Study
Source: PLoS One. 2013 Sep 3;8(9):e73047. doi: 10.1371/journal.pone.0073047 (PMC3760871; doi:10.1371/journal.pone.0073047)
Supplement: Table S2 — Episodes of exposure to maternal infection or antibiotic treatment in pregnancy. (DOCX) [file pone.0073047.s002.docx]

| **Exposure in pregnancy^a^ n (%)** | **Cases (N=1533)** | **Controls (N=14236)** |
| --- | --- | --- |
| **Antibiotic treatment** |  |  |
| No episodes | 1005 (65.6) | 10126 (71.1) |
| 1 episode | 389 (25.4) | 3026 (21.3) |
| ≥2 episodes | 139 (9.1) | 1084 (7.6) |
| **Urinary tract infection** |  |  |
| No episodes | 1351 (88.1) | 12860 (90.3) |
| 1 episode | 158 (10.3) | 1168 (8.2) |
| ≥2 episodes | 24 (1.6) | 208 (1.5) |
| **Respiratory tract infection** |  |  |
| No episodes | 1456 (95.0) | 13455 (94.5) |
| 1 episode | 70 (4.6) | 734 (5.2) |
| ≥2 episodes | 7 (0.5) | 47 (0.3) |

^a^any time from 1^st^ day of last menstrual period (LMP) to index date (for cases this is the date of pre-eclampsia, for controls this is the date they reached the same gestational age as their matched case at the case’s index date).
