# Supplementary material for: Acute Maternal Infection and Risk of Pre-Eclampsia: A Population-Based Case-Control Study
Source: PLoS One. 2013 Sep 3;8(9):e73047. doi: 10.1371/journal.pone.0073047 (PMC3760871; doi:10.1371/journal.pone.0073047)
Supplement: Table S3 — Adjusted odds ratios for early-onset (<34 weeks’ gestation) and late-onset (≥34 weeks’ gestation) pre-eclampsia. (DOCX) [file pone.0073047.s003.docx]

|  | **Matched adjusted^b^ OR (95% CI)** | |
| --- | --- | --- |
| **Exposure in pregnancy^a^** | **Early-onset pre-eclampsia (n=315 cases)** | **Late-onset pre-eclampsia (n=1218 cases)** |
| Antibiotic treatment | 1.54 (1.19-2.00) | 1.22 (1.07-1.39) |
| Urinary tract infection | 1.39 (0.94-2.06) | 1.19 (0.99-1.44) |
| Respiratory tract infection | 1.46 (0.88-2.42) | 0.81 (0.61-1.07) |

^a^any time from 1st day of last menstrual period (LMP) to index date (for cases this is the date of pre-eclampsia, for controls this is the date they reached the same gestational age as their matched case at the case’s index date).

^b^ORs adjusted for maternal age; pre-gestational hypertension, diabetes and renal disease; multifetal gestation; BMI and smoking. In addition, ORs for UTI and RTI are mutually adjusted for.
