# Supplementary material for: Acute Maternal Infection and Risk of Pre-Eclampsia: A Population-Based Case-Control Study
Source: PLoS One. 2013 Sep 3;8(9):e73047. doi: 10.1371/journal.pone.0073047 (PMC3760871; doi:10.1371/journal.pone.0073047)
Supplement: Table S4 — Results of sensitivity analyses. (DOCX) [file pone.0073047.s004.docx]

|  |  | **Matched adjusted^a^ OR (95% CI) for pre-eclampsia** | |
| --- | --- | --- | --- |
|  | **No. cases included** | **Antibiotic treatment^b^** | **UTI^b^** |
| **Primary analysis** | **1533** | **1.28 (1.14-1.44)** | **1.22 (1.03-1.45)** |
| **Sensitivity analyses, by exclusion criteria** |  |  |  |
| 1. Excluding women aged <18 years | 1489 | 1.28 (1.13-1.44) | 1.20 (1.01-1.42) |
| 2. Excluding women with pre-existing hypertension | 1371 | 1.34 (1.18-1.51) | 1.31 (1.09-1.57) |
| 3. Excluding controls with new onset hypertension in pregnancy which resolved 6-12 months after delivery | 1533 | 1.29 (1.15-1.44) | 1.24 (1.04-1.46) |
| 4. Excluding ART pregnancies | 1522 | 1.28 (1.14-1.44) | 1.21 (1.02-1.44) |
| 5. Excluding women with less than 6 months UTS data prior to conception | 1297 | 1.27 (1.12-1.44) | 1.25 (1.04-1.50) |
| 6. Excluding pregnancies before year 2000 | 646 | 1.38 (1.16-1.66) | 1.26 (0.97-1.65) |
| 7. Excluding cases with non-specific or mild pre-eclampsia | 317 | 1.29 (1.00-1.67) | 1.66 (1.16-2.39) |

Abbreviations: UTS=up-to-standard (i.e. data meeting GPRD quality standards). ART=assisted reproductive technology

^a^ORs adjusted for maternal age; pre-gestational hypertension (analyses 1, 3-7 only), diabetes and renal disease; and multifetal gestation. In addition, ORs for UTI adjusted for RTI in pregnancy.

^b^any time from 1^st^ day of last menstrual period (LMP) to index date (for cases this is the date of pre-eclampsia, for controls this is the date they reached the same gestational age as their matched case at the case’s index date).
